# Supplementary figures and images for: Type 1 diabetes mellitus patients had lower total vitamin K levels and increased sensitivity to direct anticoagulants
Source: PLoS One. 2025 Jun 23;20(6):e0326580. doi: 10.1371/journal.pone.0326580 (PMC12184912; doi:10.1371/journal.pone.0326580)

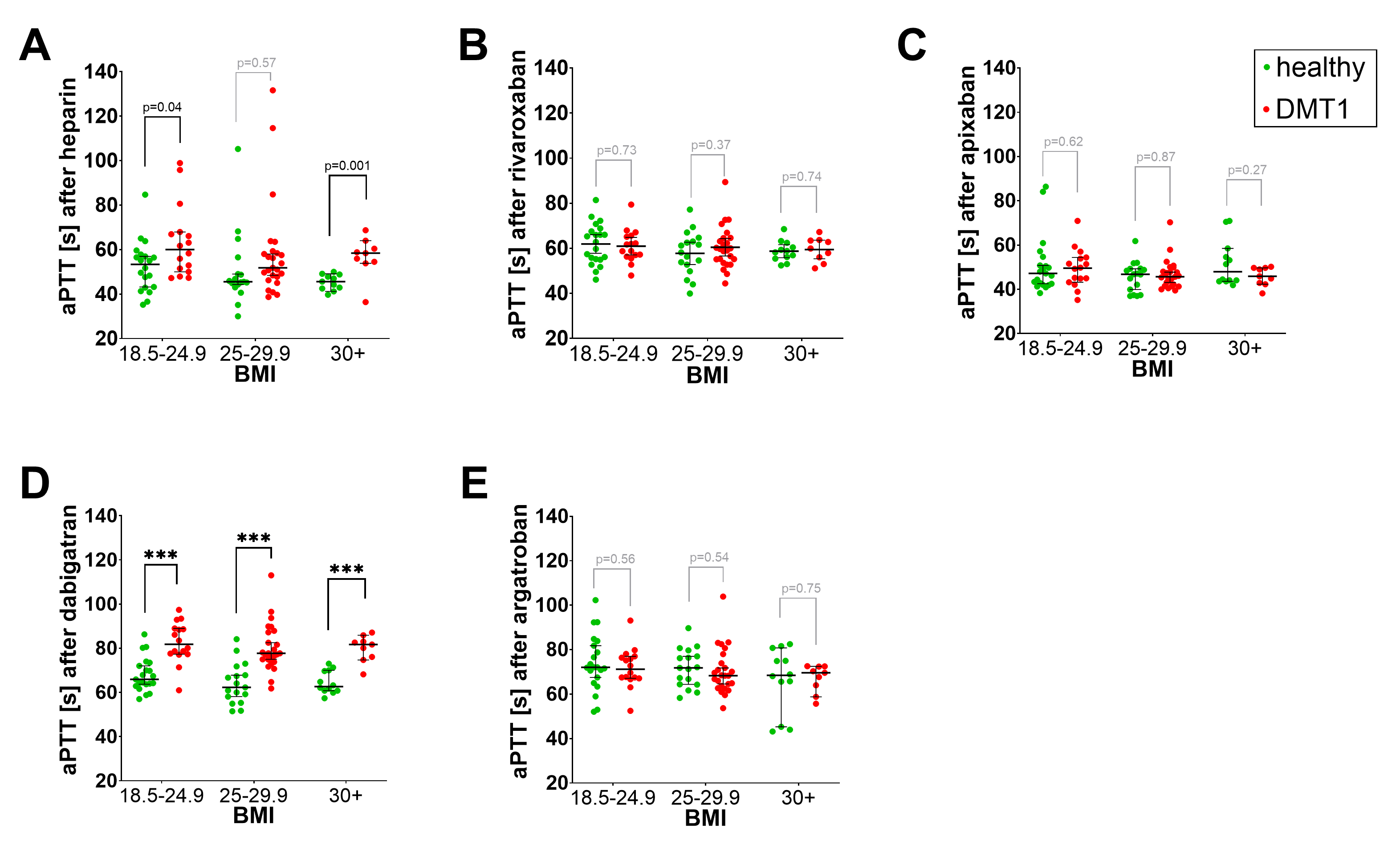

Supplement: S1 Fig — (TIF) [file pone.0326580.s001.tif]

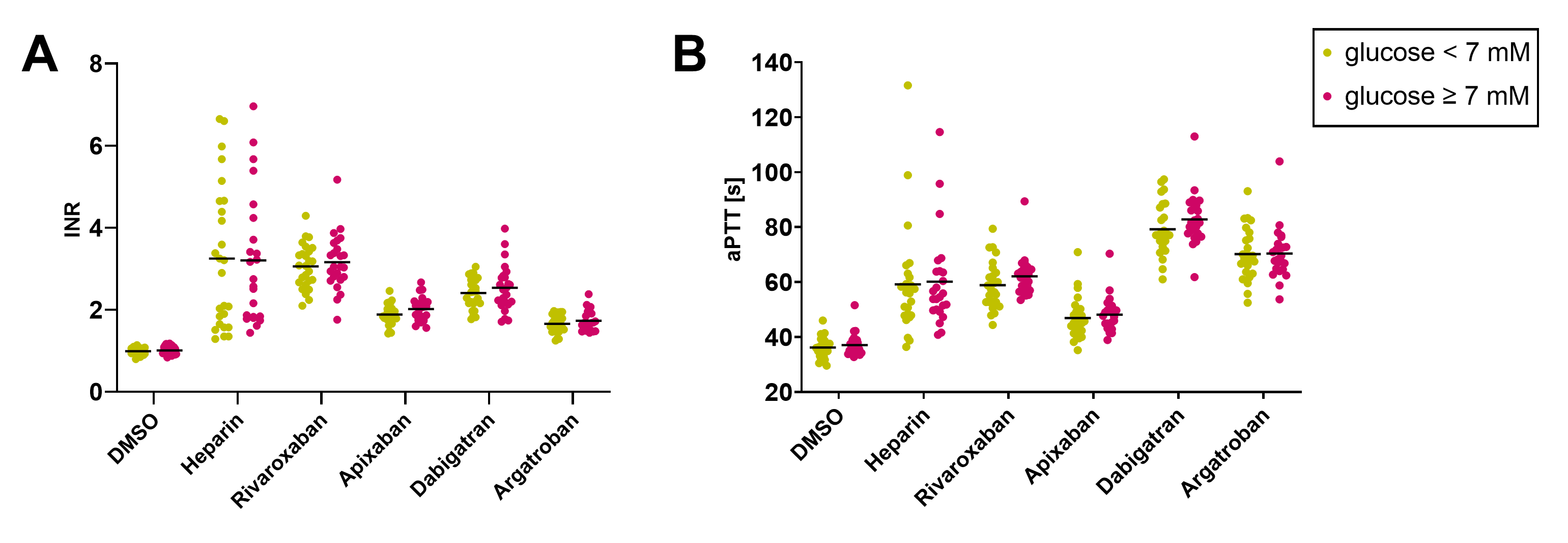

Supplement: S2 Fig — (TIF) [file pone.0326580.s002.tif]

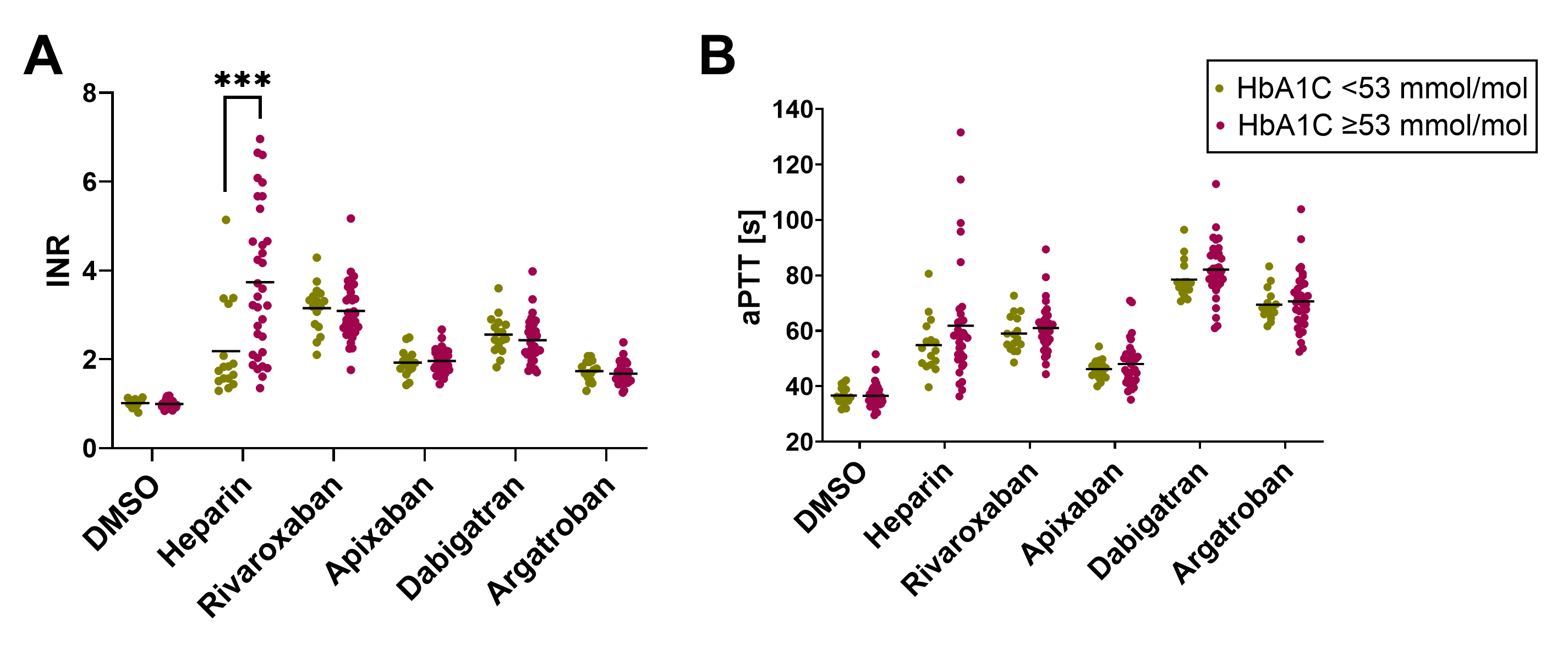

Supplement: S3 Fig — (TIF) [file pone.0326580.s003.tif]

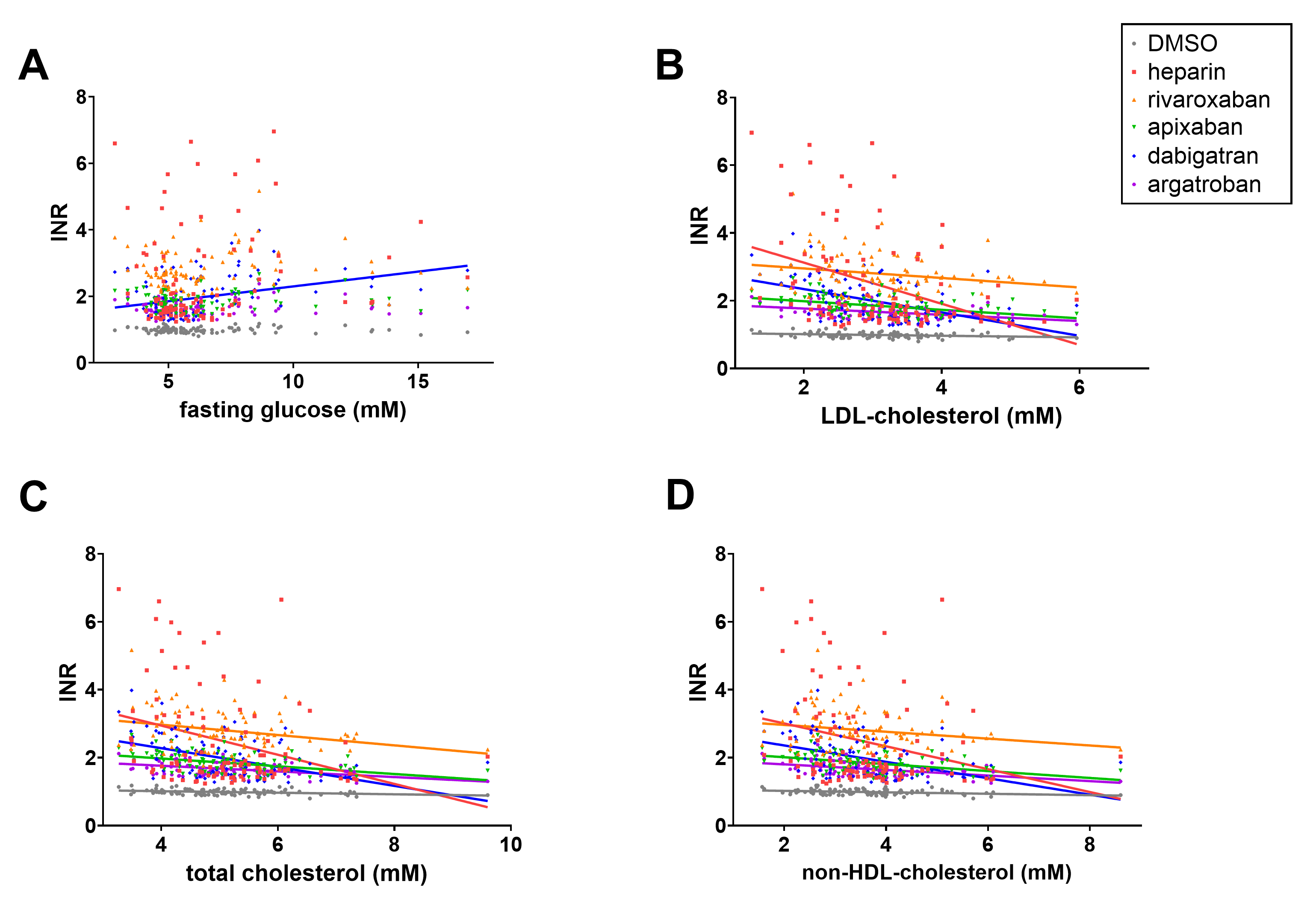

Supplement: S4 Fig — (TIF) [file pone.0326580.s004.tif]

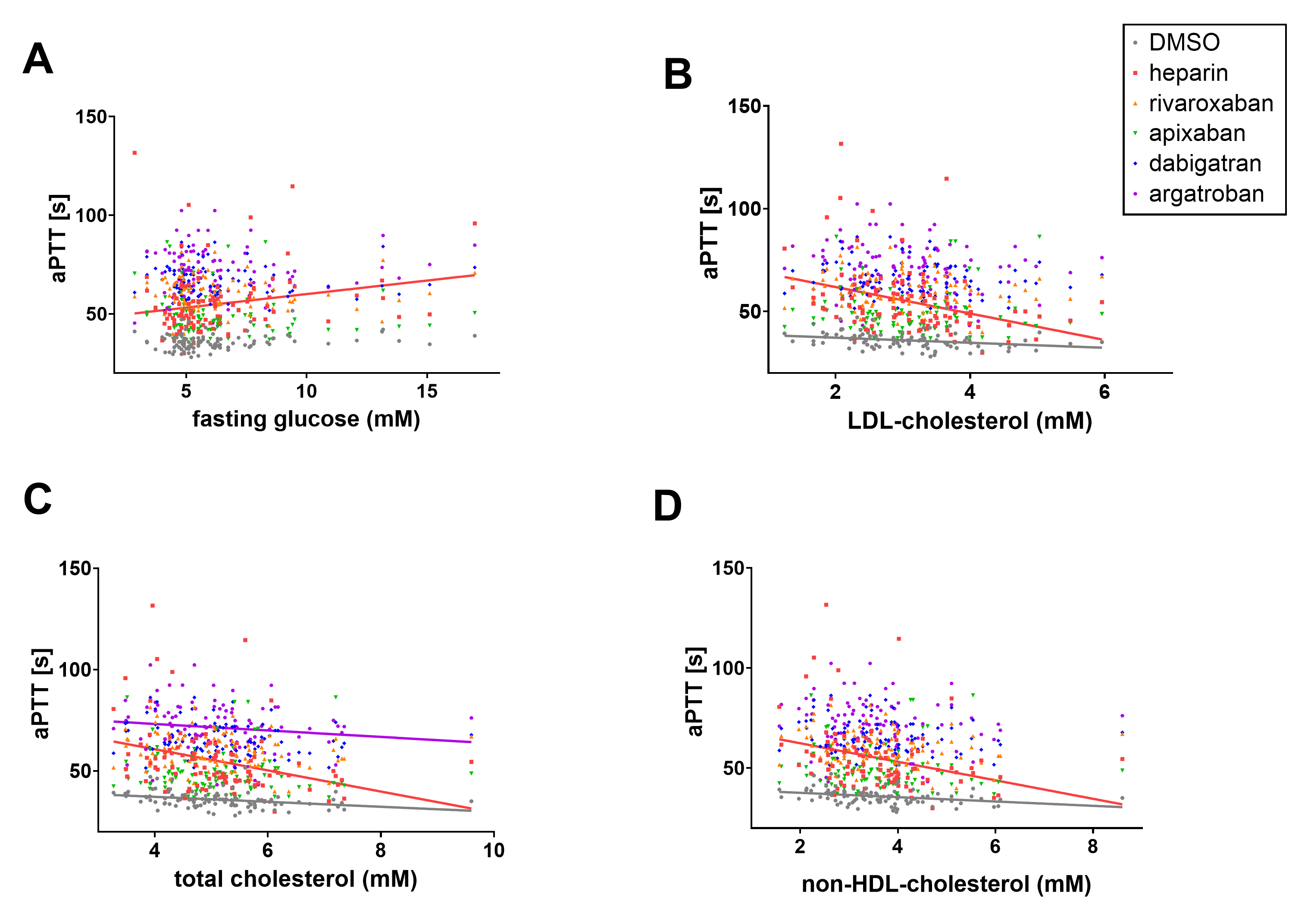

Supplement: S5 Fig — (TIF) [file pone.0326580.s005.tif]

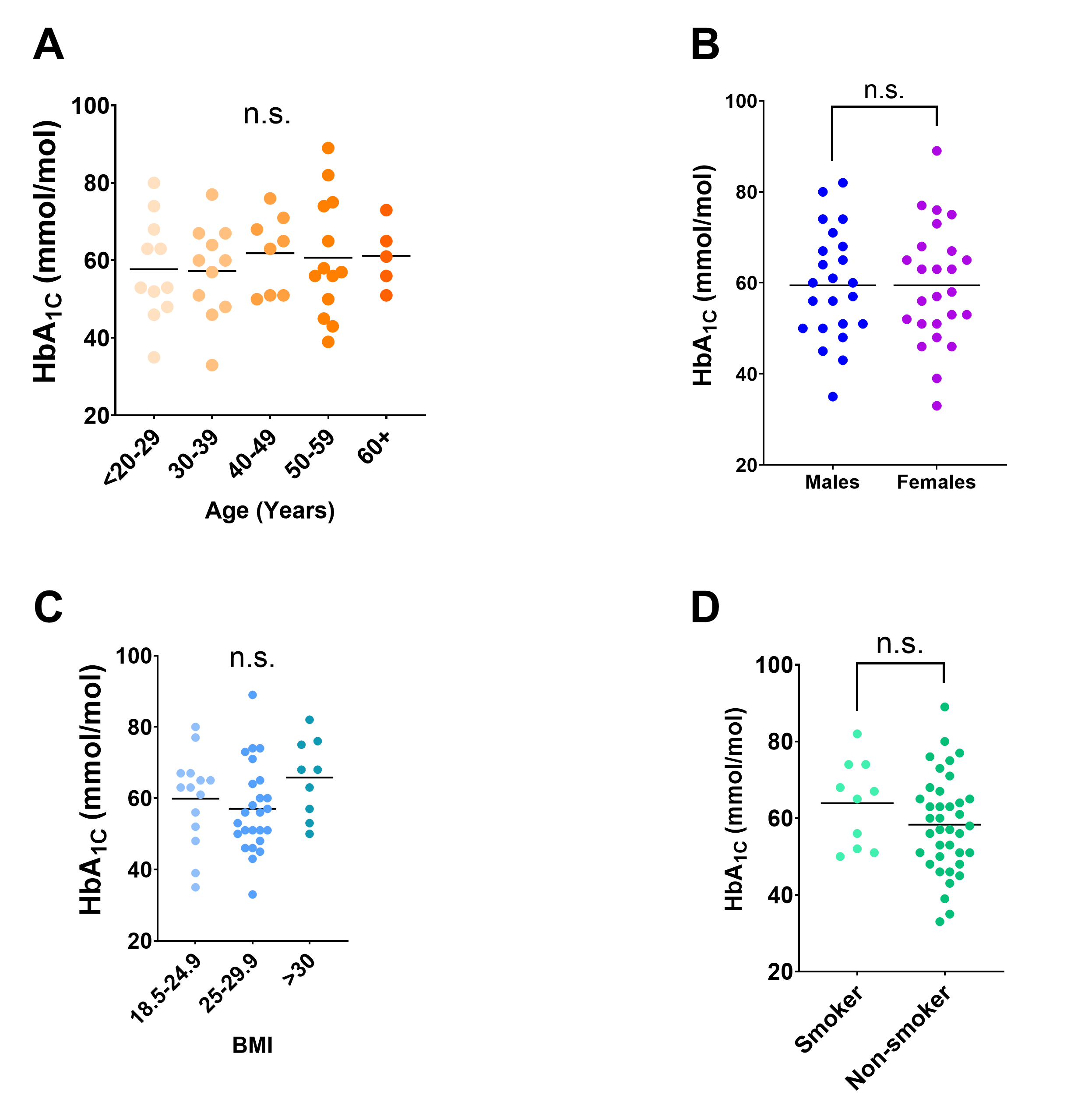

Supplement: S6 Fig — (TIF) [file pone.0326580.s006.tif]

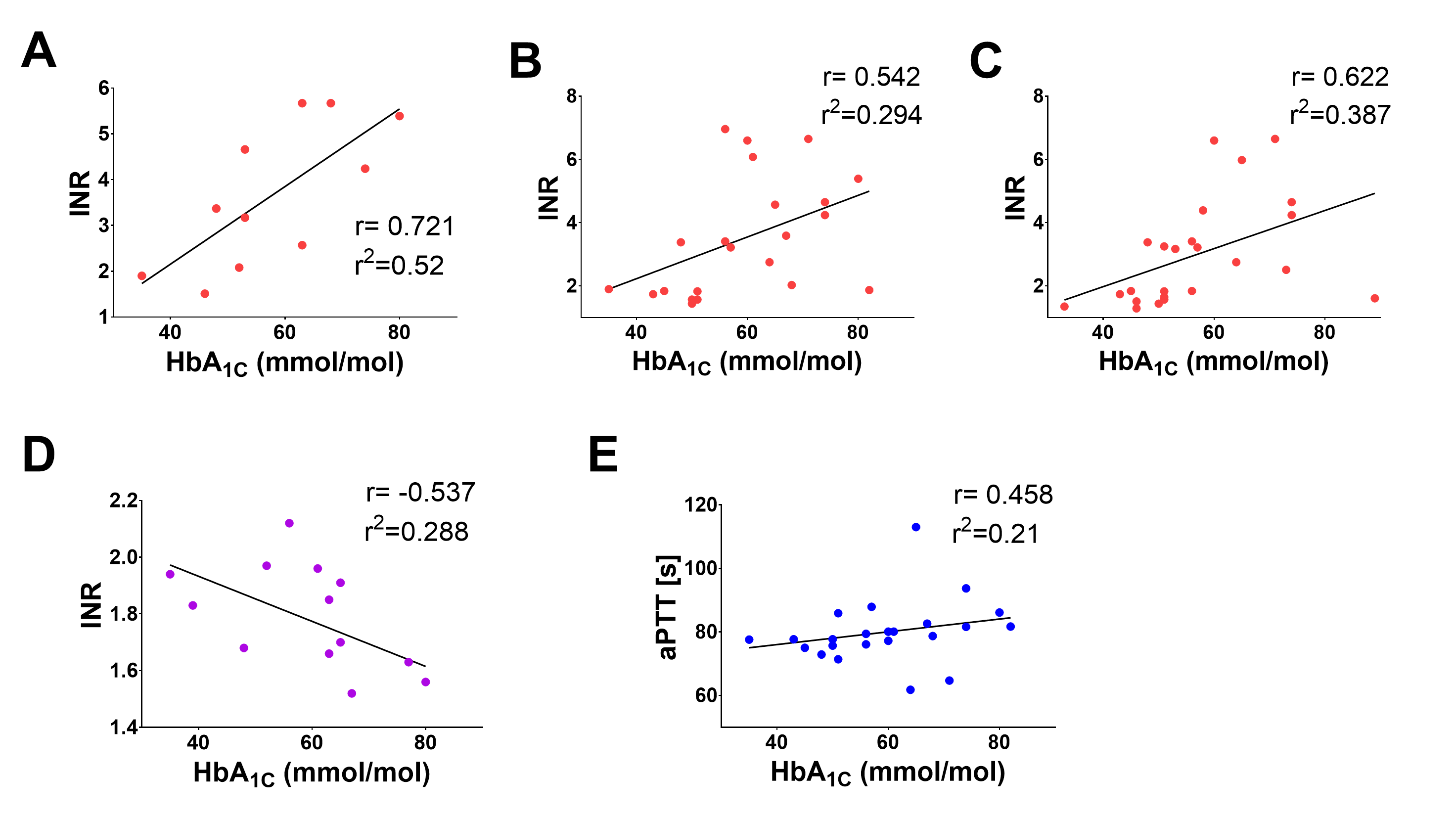

Supplement: S7 Fig — (TIF) [file pone.0326580.s007.tif]

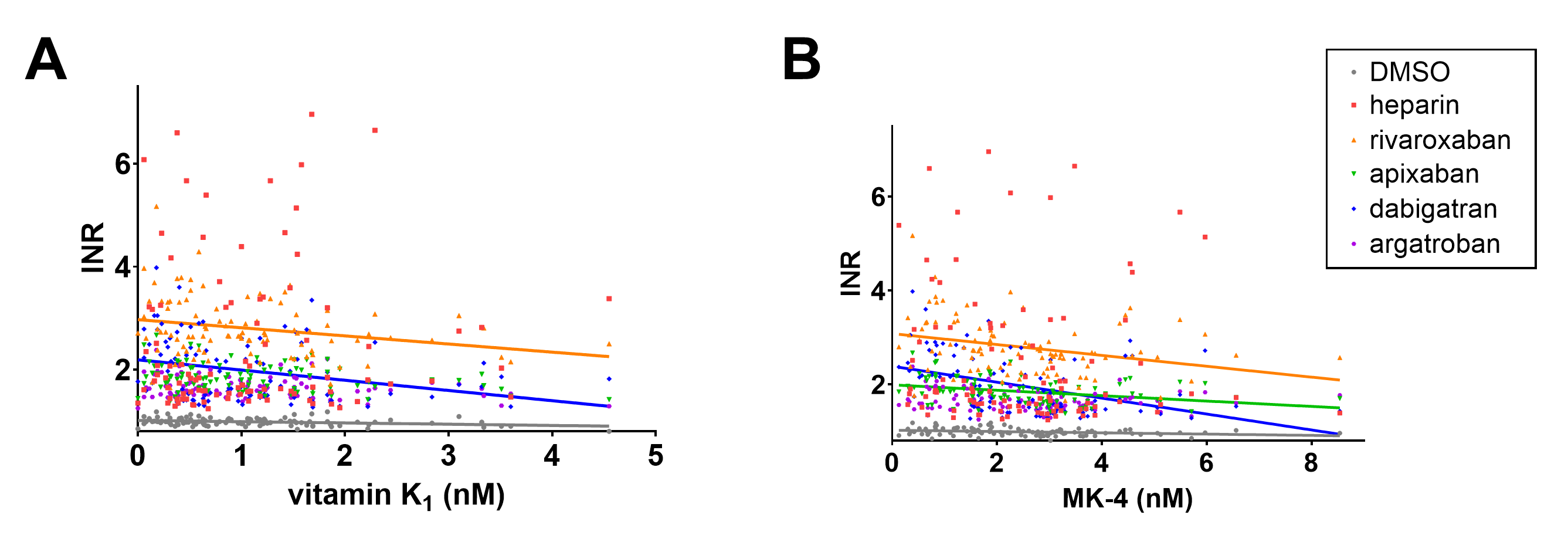

Supplement: S8 Fig — (TIF) [file pone.0326580.s008.tif]

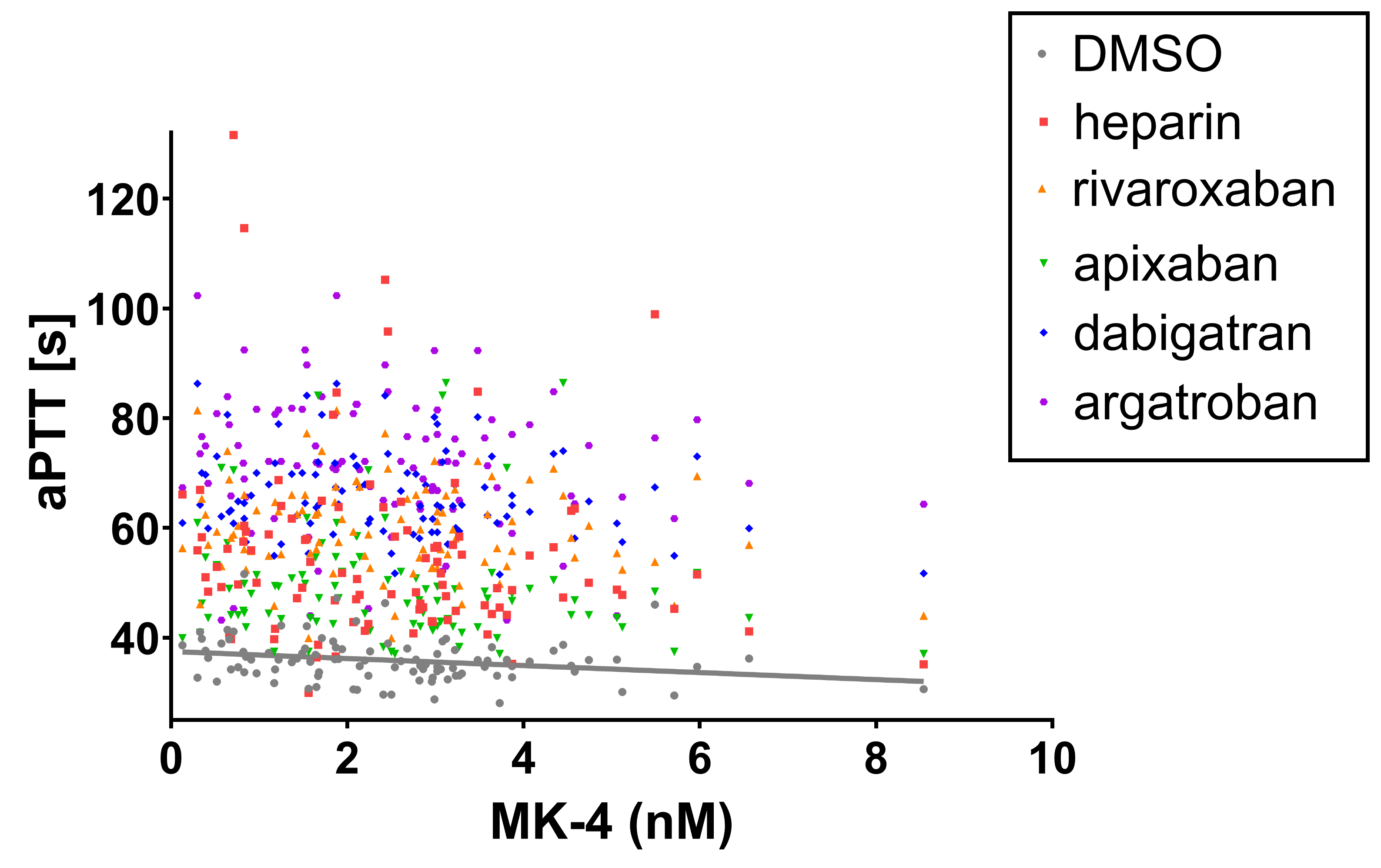

Supplement: S9 Fig — (TIF) [file pone.0326580.s009.tif]

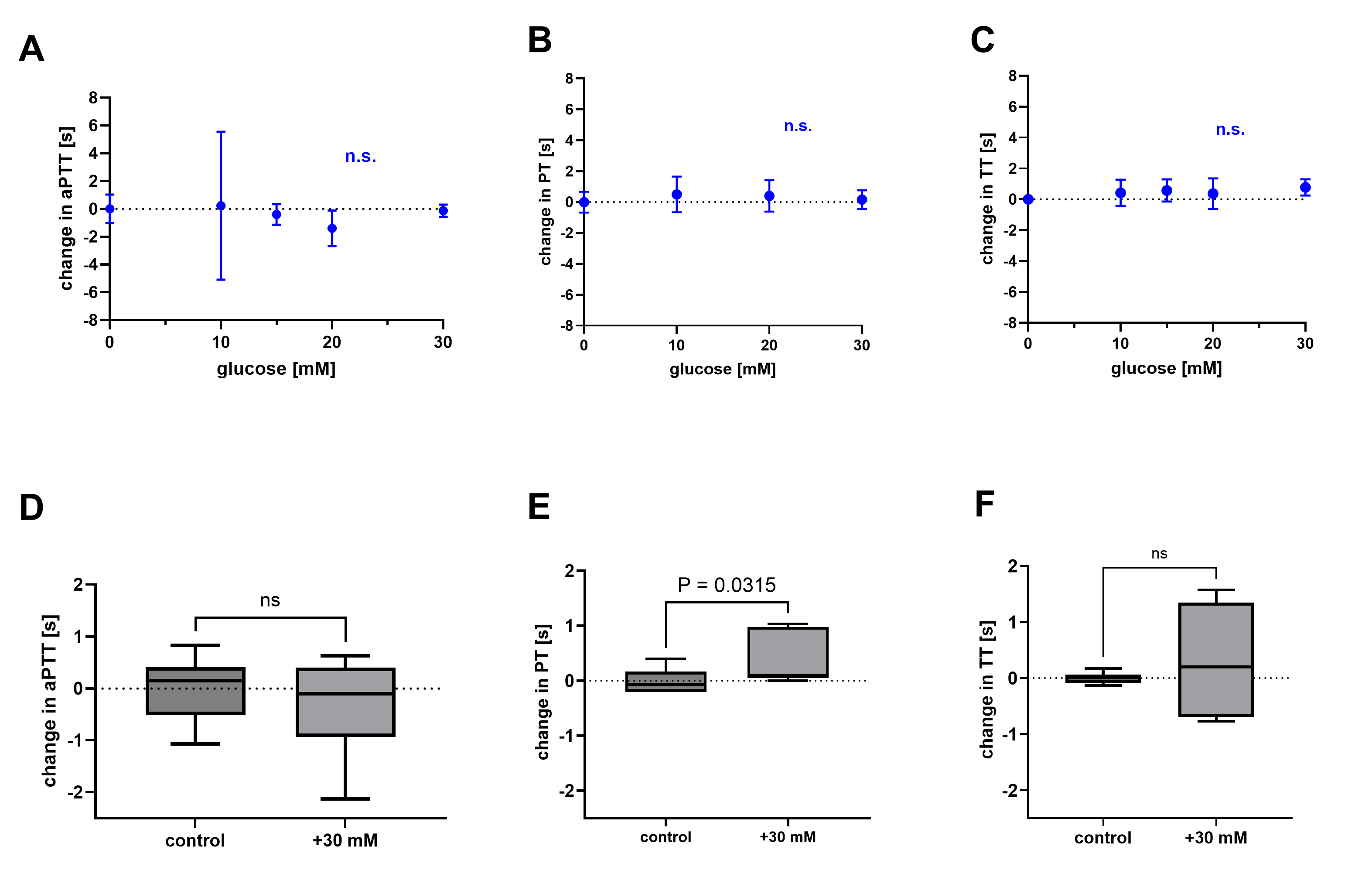

Supplement: S10 Fig — (TIF) [file pone.0326580.s010.tif]

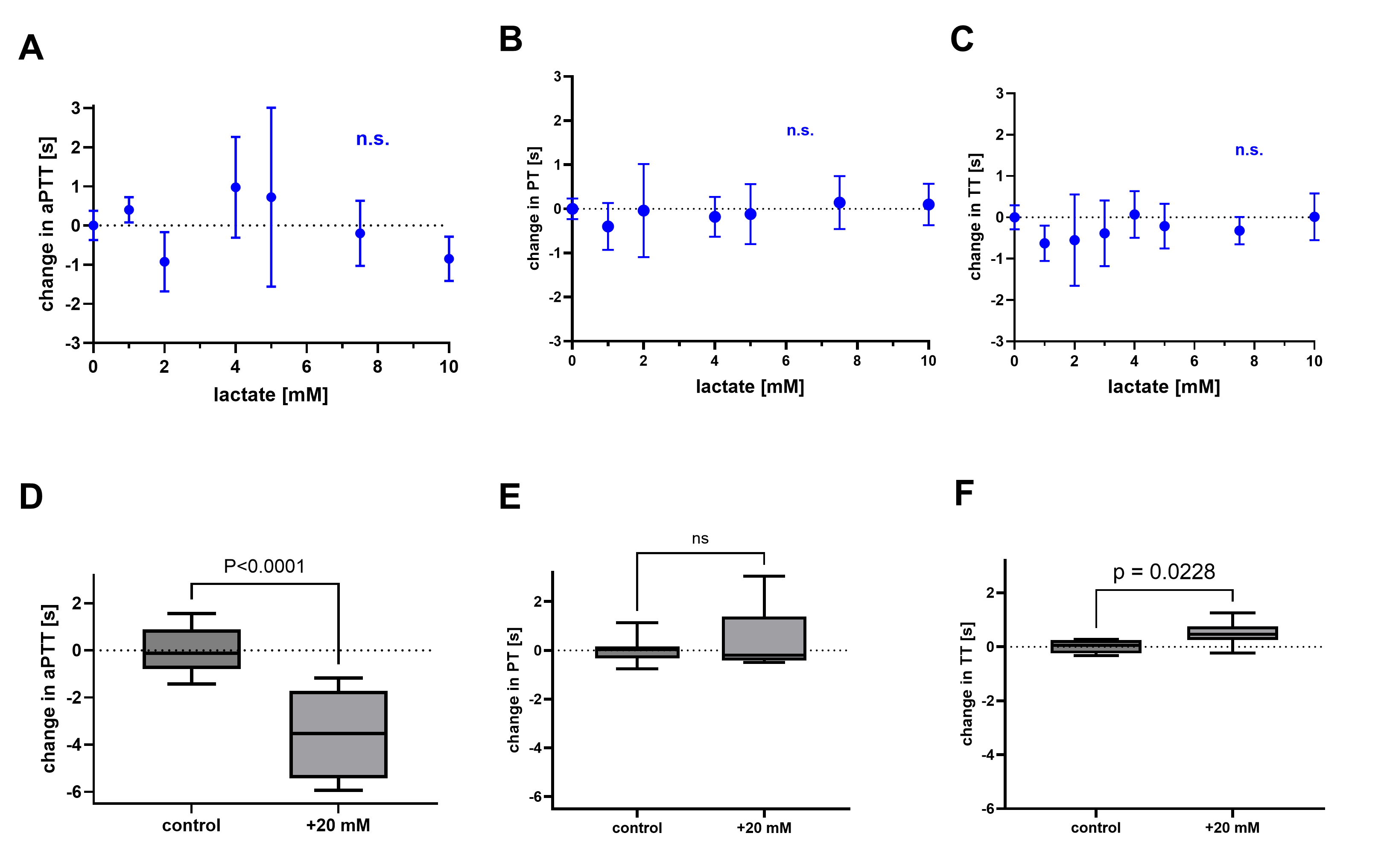

Supplement: S11 Fig — (TIF) [file pone.0326580.s011.tif]
