# Supplementary material for: Type 1 diabetes mellitus patients had lower total vitamin K levels and increased sensitivity to direct anticoagulants
Source: PLoS One. 2025 Jun 23;20(6):e0326580. doi: 10.1371/journal.pone.0326580 (PMC12184912; doi:10.1371/journal.pone.0326580)
Supplement: S1 Table — (DOCX) [file pone.0326580.s012.docx]

**S1 Table. Drugs used in generally healthy controls and patients suffering from type I diabetes mellitus**.

| **Therapy** | **Healthy** | **DMT1** | **p-value** |
| --- | --- | --- | --- |
| ACE inhibitor | **0** | **10** | **<0.001** |
| ACE inhibitor + diuretic | 0 | 1 | 0.315 |
| ACE inhibitor + Ca^2+^-channel blocker | 0 | 1 | 0.315 |
| ACE inhibitor + other antihypertensive | 0 | 2 | 0.153 |
| AT II antagonist | 5 | 3 | 0.461 |
| AT II antagonist + diuretic | **4** | **0** | **0.041** |
| β-blocker | 0 | 2 | 0.153 |
| Ca^2+^-channel blocker | 5 | 4 | 0.727 |
| Thiazide diuretic | 0 | 1 | 0.315 |
| Thiazide + potassium sparing diuretics | 1 | 0 | 0.315 |
| HMG CoA reductase inhibitor | **0** | **11** | **<0.001** |
| Fibrate | 0 | 1 | 0.315 |
| Lipid modifying combination | 0 | 1 | 0.315 |
| Glucosuric | 0 | 2 | 0.153 |
| Proton pump inhibitor | 4 | 1 | 0.169 |
| Antacid | 1 | 0 | 0.315 |
| Propulsive | 0 | 2 | 0.153 |
| Hormonal contraceptive | 5 | 5 | 1 |
| Thyroid hormone | 6 | 10 | 0.275 |
| Antibiotic | 0 | 2 | 0.153 |
| Anti-estrogen | 0 | 1 | 0.315 |
| Immunosuppressant | 0 | 1 | 0.315 |
| NSAID | 2 | 0 | 0.153 |
| Antigout | 1 | 0 | 0.315 |
| Opioid | 0 | 1 | 0.315 |
| Gabapentinoid | **0** | **7** | **0.006** |
| Antidepressant | 0 | 3 | 0.079 |
| β_2_-adrenergic agonist | 1 | 0 | 0.315 |
| β_2_-adrenergic agonist + corticosteroid | 4 | 1 | 0.169 |
| Antihistamine | **6** | **1** | **0.05** |
