# Supplementary material for: Type 1 diabetes mellitus patients had lower total vitamin K levels and increased sensitivity to direct anticoagulants
Source: PLoS One. 2025 Jun 23;20(6):e0326580. doi: 10.1371/journal.pone.0326580 (PMC12184912; doi:10.1371/journal.pone.0326580)
Supplement: S2 Table — (DOCX) [file pone.0326580.s013.docx]

**S2 Table. Statistical analysis of differences in coagulation parameters after exclusion of drug groups which were more prevalently administered either in the case of type 1 diabetes mellitus patients or healthy controls.**

|  | **exclusion of ACEi** | **exclusion of statins** | **exclusion of N03AX group*** | **exclusion of antihistamines** |
| --- | --- | --- | --- | --- |
| INR (DMSO) | p=0.21 | p=0.085 | p=0.21 | p=0.09 |
| INR (heparin) | **p < 0.001** | **p < 0.001** | **p < 0.001** | **p < 0.001** |
| INR (rivaroxaban) | **p < 0.001** | **p < 0.001** | **p < 0.001** | **p < 0.001** |
| INR (apixaban) | **p < 0.001** | **p < 0.001** | **p < 0.001** | **p < 0.001** |
| INR (dabigatran) | **p < 0.001** | **p < 0.001** | **p < 0.001** | **p < 0.001** |
| INR (argatroban) | p=0.32 | p=0.42 | p=0.51 | p=0.36 |
| aPTT (DMSO) | p=0.17 | p=0.055 | p=0.12 | p=0.07 |
| aPTT (heparin) | **p = 0.005** | **p < 0.001** | **p < 0.001** | **p < 0.001** |
| aPTT (rivaroxaban) | p=0.80 | p=0.45 | p=0.89 | p=0.71 |
| aPTT (apixaban) | p=0.75 | p=0.998 | p=0.78 | p=0.90 |
| aPTT (dabigatran) | **p < 0.001** | **p < 0.001** | **p < 0.001** | **p < 0.001** |
| aPTT (argatroban) | p=0.63 | p=0.85 | p=0.57 | p=0.85 |

*gabapentin or pregabalin (known also together as gabapentinoids)

Normality was checked by the Shapiro-Wilk. Based on normality, the Student’s unpaired t-test or the Mann-Whitney test was carried out similarly to Figure 1.
