# Supplementary material for: Type 1 diabetes mellitus patients had lower total vitamin K levels and increased sensitivity to direct anticoagulants
Source: PLoS One. 2025 Jun 23;20(6):e0326580. doi: 10.1371/journal.pone.0326580 (PMC12184912; doi:10.1371/journal.pone.0326580)
Supplement: S3 Table — (DOCX) [file pone.0326580.s014.docx]

**S3 Table. Correlation of glycated haemoglobin from DMT1 patients – subgroup analysis**

|  | **Parameters** | | **DMSO** | | **Heparin** | | **Rivaroxaban** | | **Apixaban** | | **Dabigatran** | | **Argatroban** | |
| --- | --- | --- | --- | --- | --- | --- | --- | --- | --- | --- | --- | --- | --- | --- |
|  |  |  | **r** | **p-value** | **r** | **p-value** | **r** | **p-value** | **r** | **p-value** | **r** | **p-value** | **r** | **p-value** |
| INR | Age |  |  |  |  |  |  |  |  |  |  |  |  |  |
|  |  | <20-29 | -0.435 | 0.182 | **0.721** | **0.012** | 0.06 | 0.861 | -0.168 | 0.622 | -0.384 | 0.244 | -0.523 | 0.099 |
|  |  | 30-39 | 0.065 | 0.85 | 0.349 | 0.324 | 0.174 | 0.609 | 0.186 | 0.583 | 0.048 | 0.889 | 0.192 | 0.571 |
|  |  | 40-49 | -0.51 | 0.197 | 0.551 | 0.157 | -0.341 | 0.408 | -0.154 | 0.716 | -0.241 | 0.566 | -0.482 | 0.226 |
|  |  | 50-59 | -0.092 | 0.764 | -0.197 | 0.514 | -0.044 | 0.886 | -0.294 | 0.329 | -0.407 | 0.168 | -0.298 | 0.323 |
|  |  | 60+ | -0.06 | 0.924 | -0.024 | 0.97 | 0.605 | 0.28 | 0.704 | 0.185 | 0.627 | 0.258 | 0.561 | 0.325 |
|  | Gender |  |  |  |  |  |  |  |  |  |  |  |  |  |
|  |  | Male | -0.128 | 0.559 | **0.542** | **0.009** | 0.019 | 0.933 | -0.186 | 0.396 | -0.328 | 0.126 | -0.243 | 0.264 |
|  |  | Female | -0.093 | 0.659 | 0.114 | 0.587 | -0.006 | 0.976 | 0.132 | 0.531 | -0.069 | 0.747 | -0.102 | 0.627 |
|  | BMI |  |  |  |  |  |  |  |  |  |  |  |  |  |
|  |  | 18.5-24.9 | -0.351 | 0.219 | 0.078 | 0.791 | 0.195 | 0.505 | -0.146 | 0.619 | -0.448 | 0.108 | **-0.537** | **0.048** |
|  |  | 25-29.9 | -0.068 | 0.746 | **0.622** | **0.001** | 0.09 | 0.668 | 0.022 | 0.917 | 0.076 | 0.717 | 0.11 | 0.602 |
|  |  | >30 | 0.036 | 0.926 | -0.159 | 0.684 | -0.069 | 0.86 | -0.083 | 0.833 | -0.309 | 0.418 | -0.061 | 0.875 |
|  | Smoking habit | |  |  |  |  |  |  |  |  |  |  |  |  |
|  |  | Smoker | -0.113 | 0.756 | 0.573 | 0.088 | 0.085 | 0.815 | 0.079 | 0.827 | -0.062 | 0.865 | -0.02 | 0.955 |
|  |  | Non-smoker | -0.119 | 0.478 | 0.264 | 0.114 | 0.094 | 0.573 | -0.027 | 0.87 | -0.145 | 0.385 | -0.159 | 0.34 |
| aPTT | Age |  |  |  |  |  |  |  |  |  |  |  |  |  |
|  |  | <20-29 | 0.344 | 0.301 | 0.011 | 0.975 | 0.33 | 0.322 | 0.425 | 0.193 | 0.553 | 0.078 | 0.498 | 0.119 |
|  |  | 30-39 | -0.338 | 0.307 | 0.073 | 0.833 | -0.149 | 0.663 | -0.309 | 0.356 | 0.018 | 0.958 | -0.01 | 0.976 |
|  |  | 40-49 | -0.443 | 0.273 | 0.204 | 0.632 | -0.491 | 0.221 | -0.467 | 0.246 | -0.168 | 0.694 | -0.252 | 0.55 |
|  |  | 50-59 | -0.232 | 0.446 | 0.16 | 0.6 | -0.03 | 0.923 | 0.067 | 0.828 | -0.055 | 0.857 | -0.165 | 0.589 |
|  |  | 60+ | -0.324 | 0.595 | 0.568 | 0.318 | 0.476 | 0.417 | 0.375 | 0.534 | -0.256 | 0.678 | -0.618 | 0.266 |
|  | Gender |  |  |  |  |  |  |  |  |  |  |  |  |  |
|  |  | Male | 0.002 | 0.992 | 0.304 | 0.158 | 0.113 | 0.608 | 0.07 | 0.75 | **0.458** | **0.028** | 0.214 | 0.328 |
|  |  | Female | -0.239 | 0.25 | 0.03 | 0.887 | -0.148 | 0.481 | -0.028 | 0.893 | -0.099 | 0.637 | -0.196 | 0.347 |
|  | BMI |  |  |  |  |  |  |  |  |  |  |  |  |  |
|  |  | 18.5-24.9 | 0.254 | 0.377 | 0.079 | 0.787 | 0.183 | 0.532 | 0.18 | 0.537 | 0.284 | 0.325 | 0.507 | 0.066 |
|  |  | 25-29.9 | -0.248 | 0.231 | 0.196 | 0.348 | -0.046 | 0.828 | -0.09 | 0.667 | -0.093 | 0.659 | -0.148 | 0.481 |
|  |  | >30 | -0.625 | 0.072 | 0.183 | 0.637 | -0.575 | 0.105 | -0.165 | 0.671 | -0.328 | 0.389 | -0.448 | 0.227 |
|  | Smoking habit | |  |  |  |  |  |  |  |  |  |  |  |  |
|  |  | Smoker | 0.105 | 0.773 | 0.584 | 0.082 | 0.255 | 0.477 | 0.315 | 0.375 | 0.356 | 0.313 | 0.271 | 0.449 |
|  |  | Non-smoker | -0.279 | 0.09 | -0.058 | 0.728 | -0.205 | 0.217 | -0.154 | 0.355 | -0.093 | 0.581 | -0.18 | 0.279 |
