# Supplementary material for: Type 1 diabetes mellitus patients had lower total vitamin K levels and increased sensitivity to direct anticoagulants
Source: PLoS One. 2025 Jun 23;20(6):e0326580. doi: 10.1371/journal.pone.0326580 (PMC12184912; doi:10.1371/journal.pone.0326580)
Supplement: Attachment — Submitted filename: reviewers solely.docx [file pone.0326580.s016.docx]

Answer to reviewers

**Reviewer #1**: Good paper with enough information. However there is an important issue. For these outcome measures, the sample size is so limited. Because of lots of dropouts, it was needed to have more participants. You did not mention how to estimate sample size in method section. Moreover, I did not see the limitation part.

Response: We understand and agree with the points raised by the reviewer that the number of patients is a limitation of our paper. We are aware that a population of 50 patients and 50 healthy controls is relatively small, but we have estimated sample size: The number of patients (50 in each group) should be sufficient to prove a difference of 10% in anticoagulant response (power analysis: p=0.05, power 0.95 → 26 patients in each group are needed). In fact, we have recruited 50 patients due to potential dropouts in some subanalyses. These results are a part of a screening project where we have included patients with different metabolic disorders to see if there are differences. Recruitment of more patients and healthy donors would require novel ethics committee approval (as the last one ended with the end of the project in December 2024) and probably will not bring too much novel findings (mostly there are clear differences in the coagulation effects or there are apparently no differences, e.g. Figure 1). We suppose that, given the observed differences, the number of recruited blood donors was sufficient for this publication. Regardless, we newly specified power analysis in our paper (in the chapter on statistical analysis), and we have also added a new chapter on limitations.

**Reviewer #2**: This manuscript, titled "Type 1 Diabetes Mellitus Patients Had Lower Total Vitamin K Levels and Increased Sensitivity to Direct Anticoagulants," explores an intriguing and clinically significant intersection between diabetes mellitus, coagulation pathways, and the efficacy of anticoagulants. The study aims to shed light on potential differences in anticoagulant sensitivity between patients with type 1 diabetes mellitus (DMT1) and healthy individuals, while also examining the role of vitamin K levels in mediating these differences. The research is notable for its direct comparison of different classes of anticoagulants and its attempt to correlate biochemical markers with coagulation outcomes. However, the manuscript, as submitted, has several areas that warrant substantial revision. Addressing these issues will not only enhance the scientific rigor of the work but also its potential impact on clinical practice and public health.

Response: We are thankful for a very detailed assessment of our paper as well as for encouraging comments. Below are detailed answers to all comments.

The following detailed review outlines specific recommendations for improving the manuscript:

Abstract
p.2, lines25–46:
The abstract lacks a concise statement of the clinical or public health relevance of the findings. How these findings might influence current clinical practices is not well articulated. Terms like "paradoxically better lipid profiles" need clarification; such phrasing is ambiguous without context. The results are described without confidence intervals, p-values, or other statistical measures, which are essential to substantiate claims.

Response: Thank you for this important feedback that our abstract was not sufficiently succinct. We have modified the abstract according to your suggestions and the recommendation of reviewer No.4. We believe that the abstract, which is a compromise between reviewer comments and the space limitation, is stronger now.

Introduction
p. 3, lines 61–66:
The introduction does not sufficiently highlight gaps in existing literature to justify the study’s necessity. It assumes the audience is already aware of the limitations in current research on DMT1 and anticoagulation.

Response: Thank you for pointing out this gap. This part was indeed not emphasized, we have modified it to underline the need for such a study.

p. 3, lines 66–70:
Statements regarding the suitability of direct anticoagulants for DMT1 patients are broad and unsupported by specific prior studies or references.

Response: Thank you for this comment, as the previous version of the article was not written clearly enough, as there are no clear previous indices that direct anticoagulants should be used in preference in the diabetic population over other patients (e.g., replacement arthroplasty). We have modified that part, searched for available literature, and added clinically relevant data, and also one relevant experimental paper.

p. 3, lines 78–90:
The description of coagulation assays lacks precision. Specific references to how these tests uniquely contribute to the study goals could strengthen the argument.

Response: Thank you for this comment, which was also similar the reviewer No.3. It was quite unexpected that, notwithstanding these tests are frequently used, their relationship to cardiovascular morbidity and mortality has not been extensively reported. We found two relevant papers, which are mentioned in the introduction.

Methods
p. 5, lines 99–102:
No rationale is provided for choosing 50 participants per group. Was a power calculation conducted to ensure adequate sample size?

Response: Yes, we have estimated sample size: The number of patients (50 in each group) is sufficient to prove a difference of 10% in anticoagulant response (power analysis: p=0.05, power 0.95 → 26 patients in each group are needed). There is an additional important aspect. These results are part of a larger project where we screened the differences between different patients suffering metabolic diseases; hence, in order to make such a project feasible, we also calculated the number of patients also with regard to funding limitations. We have added information about power analysis in the statistical section of the paper.

p. 5, lines 101–105:
The criteria are described but lack justification. For instance, why were individuals with hypothyroidism or hypertension included if these conditions could influence coagulation?

Response: Thank you for this important comment. In principle, it is not easy to have a healthy control group encompassing a population with a large age range from 20 up to 60 years, where all donors will be fully healthy. Therefore, we had to accept some conditions, such as well-treated hypothyroidism and hypertension. Given the relatively frequent incidence of these conditions in the general Czech population, we considered it acceptable for inclusion. We have also added this information in the revised version of the paper.

Control Selection:
The control group, described as “generally healthy,” is not defined rigorously. Are they comparable to DMT1 patients in lifestyle and diet?

Response: This is an important point. In principle, diabetic persons should follow some recommendations about lifestyle and diet. It might not be easy to monitor them. The common food questionnaires are based on patient self-reporting and patients might not be fully sincere. In our „generally healthy“ population, we did not require specific diet or lifestyle modification. Our aim was to include a real population in real-life conditions. Anyway, we have stored some plasma at -80°C in which we will measure metabolites of (food) polyphenols. Unfortunately, the method for their detection is very ambitious and our colleagues from the Analytical department have not yet fully validated the method. So we cannot report these data at the moment. We have, however measured tryptophan in the serum, which might give us some outlook if there are differences. Indeed, there was a difference; serum level of tryptophan was 66.62 ± 10.08 μM in healthy donors and 58.69 ± 13.09 μM in DMT-1 patients (both data had Gaussian distribution, t-test analysis, the difference was significant at p=0.001). In addition, we measured some inflammatory markers. See, please, our new version of the paper, where we included these data. These markers are not specific to lifestyle but might give another outlook as they do not seem to be directly linked to other risk factors like LDL-cholesterol (our new Table 3).

Confounding Variables:
The study fails to address the influence of potential confounders such as inflammatory markers, diet, or medication use.

Response: As mentioned above, we measured some relatively newer inflammatory markers with a well-documented cardiovascular relevance (neopterin and the ratio kynurenine to tryptophan). We thought to keep these data for another publication, but based on the reviewer's comment, we added the data in the publication. We are not sure if the inflammation is a confounder, based on the aim of this study, we rather think that this is another risk factor of cardiovascular disease. Concerning diet, we can perform some analysis later when we have all the data. We apologize, but we cannot assess the relationship now. We mentioned it as a limitation of our study. Medication can definitely represent a confounder. We looked at drugs which were unequally distributed between generally healthy donors and diabetic patients (Table S1: ACE inhibitors, angiotensin II antagonists, HMG CoA reductase inhibitors, gabapentinoids and antihistamines) with exception of insulin which was logically given to all patients but not to healthy person and hence its impact cannot be drawn from our study. A new table (S2 Table in the supplementary file) based on these results was prepared. As the results were almost similar (there were no differences in significance), we suppose that these drugs have not influenced our results.

References:

Avanzas P, Arroyo-Espliguero R, Kaski JC. Neopterin and cardiovascular disease: growing evidence for a role in patient risk stratification. Clin Chem. 2009;55(6):1056-7. <https://pubmed.ncbi.nlm.nih.gov/19395434/>

Baumgartner R, Forteza MJ, Ketelhuth DFJ. The interplay between cytokines and the Kynurenine pathway in inflammation and atherosclerosis. Cytokine. 2019;122:154148.

<https://pubmed.ncbi.nlm.nih.gov/28899580/>

Teunis CJ, Stroes ESG, Boekholdt SM, Wareham NJ, Murphy AJ, Nieuwdorp M, Hazen SL, Hanssen NMJ. Tryptophan metabolites and incident cardiovascular disease: The EPIC-Norfolk prospective population study. Atherosclerosis. 2023 Dec;387:117344 <https://pubmed.ncbi.nlm.nih.gov/37945449/>

Wang Q, Liu D, Song P, Zou MH. Tryptophan-kynurenine pathway is dysregulated in inflammation, and immune activation. Front Biosci (Landmark Ed). 2015;20(7):1116-43. <https://pubmed.ncbi.nlm.nih.gov/25961549/>

Results
p. 8–12:
The narrative description of results omits some key statistical details. For example, confidence intervals for effect sizes are not reported.

Response: Thank you for pointing this out. Instead of adding key statistical details directly in the text, we have supplemented the missing information directly in the tables and graphs. We think that this is a better solution as the interested reader can find them in the paper, but many details in the text might render the part of the results less readable.

The table (1) with results was not completely clear in terms of statistical analyzes. We re-checked all data; in most cases, the data did not have a Gaussian distribution, so they are reported currently as median and 95% confidence intervals. In case of the Gaussian distribution, we reported the data as mean ± SD.

p. 9–12:
There is minimal discussion of non-significant findings. This could introduce bias in interpreting the data.

Response: We understand this point and we agree that some non-significant results should also be discussed. We modified the discussion by the addition of other points based on your and other reviewer comments. Logically, due to the fact that our paper produced a large dataset, not all insignificant findings could be discussed. Current discussion already has 7 pages so we think that additional prolongation will rather be counterproductive.

Ambiguity in Causality:
Correlations between vitamin K levels and coagulation outcomes are mentioned, but causation is implied without robust evidence.

Response: This is also a very good point. As far as we know, we are the first to measure different forms of vitamin K in patients and to correlate them also with coagulation. The physiology of vitamin K is very complicated (e.g. our recent paper on vitamin K in Nutr Rev or even very fresh papers on vitamin K in relation to inflammation, Lacombe et al., Xie et al.). Even if we could not bring some conclusive data at the moment, we have discussed this issue in more detail. Also, the addition of inflammatory markers to this paper increased the informative value of this paper.

Reference:

Lacombe J, Ferron M. Vitamin K-dependent carboxylation in β-cells and diabetes. Trends Endocrinol Metab. 2024;35(7):661-73 <https://pubmed.ncbi.nlm.nih.gov/38429160/>

Mladěnka P, Macáková K, Kujovská Krčmová L, Javorská L, Mrštná K, Carazo A, Protti M, Remião F, Nováková L; OEMONOM researchers and collaborators. Vitamin K - sources, physiological role, kinetics, deficiency, detection, therapeutic use, and toxicity. Nutr Rev. 2022;80(4):677-698. <https://pubmed.ncbi.nlm.nih.gov/34472618/>

Xie Y, Li S, Wu D, Wang Y, Chen J, Duan L, et al. Vitamin K: Infection, Inflammation, and Auto-Immunity. J Inflamm Res. 2024;17:1147-60 <https://pubmed.ncbi.nlm.nih.gov/38406326/>

Demographic Stratification:
While stratified analyses by age and BMI are conducted, their clinical implications are not clearly discussed.

Response: Similarly to a previous comment by the reviewer, we find it very relevant. It is not very simple to discuss these data with a relatively limited number of patients in particular with higher age. Regardless, as we included inflammatory markers, they helped us to understand at least partly the relationship. We discussed these aspects at least in part in our new discussion.

Discussion
p. 14–17:
Claims regarding the protective effects of lower cholesterol and vitamin K levels in DMT1 lack robust evidence. The discussion overstates the findings’ implications.
Confounding Factors:
The potential influence of unmeasured variables (e.g., inflammatory states or micronutrient deficiencies) is not adequately acknowledged.

Response: The reviewer is correct that our data cannot be fully conclusive given the limited number of patients and a limited number of tested parameters. Concerning inflammation, this can definitely affect blood hemostasis. We have added new data which shows a relationship between inflammation and responses to the most active direct anticoagulants. From micronutrients, we assessed solely vitamin K as it has a direct role in blood coagulation. We were not able to test other micronutrients due to their high numbers. Regardless, we have included a limitation section in our paper, where we emphasized the possible dietary differences.

Comparison to Prior Studies (p. 14):
The discussion does not sufficiently critique or compare the study's results with contradictory findings from other research.

Response: We apologize, we tried our best to find relevant papers on the topic. In this improved version of our paper, also in relation to the comments of other reviewers, we have added more papers. If the reviewer still thinks that some specific additional paper(s) should be discussed, please let us know.

Limitations (p. 17):
Limitations are inadequately addressed. For example, no mention of the small sample size or lack of long-term follow-up is made.

Response: We agree with the need to better address the limitations of our paper. We have included this aspect in the new version of our manuscript.

p. 18:
The conclusion is overly broad and lacks actionable insights for clinical practice.

Response: Establishing a clear clinical outcome from our results is not simple, but we have carefully modified the conclusion section in relation to this aspect.

**Reviewer #3:** The authors performed an interesting study. There are potential issues in methodology and thus interpretation of results. Specifically, I have the following comments:
1. I am not sure whether PT/APTT are regularly used to assess the prothrombotic burden

Response: Both PT and aPTT are methods used in clinical practice to address the suitability of the anticoagulant therapy in patients treated with warfarin or heparins. Modern anticoagulants such as thrombin inhibitors or Fxa inhibitors are safer and are generally considered not to need monitoring. The reviewer is right that PT/aPTT are not commonly used for monitoring the effect of direct anticoagulants. Anyway, based on our data, these parameters are useful research tools that points out clear differences in the effect of these drugs. Concerning cardiovascular risk, the relationship of PT/aPTT to cardiovascular morbidity and mortality has not been extensively reported, notwithstanding frequent use of these tests. We found two relevant papers which are now shortly mentioned in the introduction.

2. Similarly, PT/APTT may not be the standardised way to measure the anticoagulation effect of DOACs

Response: The reviewer is right. As mentioned in point 1, modern anticoagulants are safer than their older “siblings”, and generally do not need to be monitored in clinical practice. We use these methods to enable an easy comparison between direct thrombin and Xa inhibitors.

3. Similarly, APTT instead of PT should be used to measure the effect of heparin

Response: The reviewer is right that heparin is more sensitive to aPTT than to PT. In fact, in our experiments, we used different concentrations of heparin (final concentrations of 5 and 0.5 IU/mL for PT and aPTT assays, respectively). Heparin was used in this study as the positive control, we did not intend to use it as one of the investigated drugs since it is now rarely used clinically (e.g., cumbersome in pharmacokinetics and burdened by some hardly predictable side effects).

4. As the authors have noted, it was quite surprising that DMT1 individuals had lower levels of total cholesterol and LDL. Any possible explanation?

Response: Patients suffering metabolic diseases, particularly DMT1 patients, with an early disease onset, should generally be subjected to a stricter diet, but more importantly, they are controlled more frequently by physicians than the generally healthy population. Indeed, looking at the medication they are taking, we suppose that the cholesterol levels are more tightly controlled in these patients than in healthy controls. In the Czech Republic, the presence of (saturated) fats in the diet and alcohol consumption is particularly high. We suppose these are some of the most evident reasons behind this surprising outcome. We also specified it newly more concretely in the discussion.

5. It was unclear why the authors performed subgroup analysis according to BMI. More elaboration is needed

Response: In principle, overweight and obese persons present a higher risk of developing dyslipidemia and diabetes mellitus. A proinflammatory state is frequently associated with these diseases, and hence, they have a higher cardiovascular risk. For this reason, we studied BMI-coagulation results since we were interested if 1) coagulation can be affected by higher BMI ex vivo (i.e., differences in distribution volume are excluded), and 2) the effects of anticoagulants are also dependent on BMI. BMI ranges were chosen based on the international classification.

6. Did the authors perform analysis correlating the strength of (anti-)coagulation and markers of insulin resistance?

Response: This is an interesting point. In principle, we included patients with type 1 diabetes mellitus and generally healthy controls, so the question of insulin resistance should not be the major issue (at least in younger patients) in comparison to type 2 diabetes mellitus, where insulin resistance is the key factor. For this reason, we have not measured insulin resistance, but we concentrated on other more common biochemical markers in type 1 diabetes mellitus patients. We also added data on inflammatory markers, which we also measured (see please also the answers to other reviewers).

7. Were there studies reporting an association between shortened PT/APTT and atherosclerotic risk?

Response: Thank you for this comment, which was also similar to the reviewer No.2. We were able to answer this question together with your question No. 1; please see the response above.

8. Testing of anticoagulant level seems clinically more relevant than testing the effect at equimolar concentrations

Response: We understand the point of view of the reviewer. We included references pointing out that the concentrations used in this study were clinically relevant, i.e., can be detected in plasma from patients being treated with these drugs. Moreover, our purpose was to compare the effectiveness of these anticoagulants in real samples. Therefore, we supposed that it would be more scientifically sound to test all drugs at the same concentrations. We should also mention that there are apparently differences in plasma levels of different anticoagulants in various patients (e.g. Godino et al., Eur Heart J 2023; Pfrepper et al., Res Pract Thromb Haemost 2020; Siedler et al., J Thromb Haemost 2022) so still a selection of the tested concentration would suffer from this drawback.

References:

Godino C, Cozzani G, Nemola G et al. Plasma levels of direct oral anticoagulants in non-valvular atrial fibrillation patients at the time of acute cardioembolic or bleeding events, Eur Heart J 2023; 44(Supplement2): ehad655.546

Pfrepper C, Metze M, Siegemund A, Klöter T, Siegemund T, Petros S. Direct oral anticoagulant plasma levels and thrombin generation on ST Genesia system. Res Pract Thromb Haemost. 2020;4(4):619-627.

Siedler G, Macha K, Stoll S, Plechschmidt J, Wang R, Gerner ST, Strasser E, Schwab S, Kallmünzer B. Monitoring of direct oral anticoagulants plasma levels for secondary stroke prevention. J Thromb Haemost. 2022;20(5):1138-1145.

9. The authors may consider performing a mediation analysis to support the findings

Response: We are deeply sorry, but we do not understand which type of analysis the reviewer is thinking of.

10. Please elaborate more on potential clinical implications and limitations

Response: In line with the comments of the reviewer No.2, we have added a limitation section to our manuscript as well as modified the conclusions with a clinical outlook.

**Reviewer #4**:

1. Grammatical and Orthographic Review:
The manuscript is largely well-written with minor grammatical issues and awkward phrasing in some places. These areas could benefit from small revisions for clarity and flow:
Abstract: The sentence "There is, however, no effect comparison among different direct anticoagulants" could be more clearly phrased as "However, no comparative studies have been conducted on the effects of different direct anticoagulants."
Introduction: The transition from general coagulation background to diabetes is slightly abrupt. A smoother transition, linking diabetes-specific coagulation issues with broader coagulation principles, would improve clarity.
Methods: The description of chemical treatments and drug concentrations is concise, but the sentence "Heparin and dabigatran treatment resulted in longer coagulation in DMT1 when compared to healthy individual in both tests" can be improved to "Heparin and dabigatran treatments resulted in prolonged coagulation in DMT1 patients compared to healthy individuals in both tests."
Figures and Tables: Ensure that figure legends are consistently formatted. For example, "heparin (final concentration of 5 IU/mL)" could be "Heparin (5 IU/mL final concentration)" for consistency.
Results: In the sentence "There were however clear differences in vitamin K levels," adding a comma after "were" would enhance readability: "There were, however, clear differences in vitamin K levels."

Response: We are thankful for the careful reading by the reviewer. All suggestions were considered and accepted, and the manuscript has been modified accordingly.

2. Scientific Rigor:
Design and Methodology: The study is well-designed, using both control and experimental groups, and applies standard biochemical methods. However, it could benefit from more detailed information regarding the statistical tests used for each analysis. For example, when reporting results from the Student's t-test or the Mann-Whitney test, it's important to note the specific assumptions or conditions under which each test was applied, particularly since they differ in handling data distribution.
Cohort: The study is limited to a sample size of 50 DMT1 patients and 50 healthy controls. While this is a reasonable sample size for pilot studies, larger cohorts may be required to validate the findings robustly and examine variability across different subgroups (age, gender, comorbidities).

Response: We fully agree with this comment. As mentioned also in answers to other reviewers, this is a pilot study in which we aimed to complexly map the coagulation response in a broad spectrum of patients with metabolic disorders. In line with comments from other reviewers, we have discussed it accordingly in the limitation section. The reviewer is right that Table 1 was somehow confusing; we have re-checked all data, which are now more clearly reported. In most cases, the data did not have a Gaussian distribution, therefore, they are currently reported as median and 95% confidence intervals. In cases of the Gaussian distribution, we reported the data as mean ± SD.

Data Presentation: The data is presented well in tables and figures but ensuring that all statistical analyses are well-documented in the methods section (for reproducibility) is crucial. The authors might also consider adding confidence intervals for their estimates, which would provide more transparency and insight into the statistical reliability of their findings.

Response: We have added missing information on statistical analysis to graphs and tables. Also, 95% confidence intervals were added to Table 1 and the relevant Figures.

Vitamin K Levels: The study finds that DMT1 patients have lower total vitamin K levels compared to healthy controls, which is an intriguing result. However, the authors could further discuss the possible mechanisms behind this, referencing other literature on vitamin K and diabetes, especially in terms of bioavailability or altered metabolic pathways in DMT1 patients.

Response: This is a very good point. However, we are afraid that we do not have a clear answer to this question. As far as we know, we are the first to report data on serum levels of 4 forms of vitamin K in healthy donors and DMT1 patients. There might be, in principle, differences in absorption, distribution, use, and elimination. Distribution is likely not involved as vitamin K is mainly present in HDL fraction, and our DMT1 patients present very similar lipoprotein levels as healthy donors. On the other hand, we added novel data on inflammatory markers, and there was a negative relationship between total vitamin K, MK-4, and K_1_ forms with these markers. From a simple correlation analysis, no causality can be detected, but there are two possible explanations: either vitamin K is highly consumed in diabetic patients due to an inflammatory state, or lower vitamin K might facilitate the inflammation. These hypotheses need to be tested in a different model, for example, in rats.

Regardless, there are novel papers suggesting the role of vitamin K in controlling glycaemia. We have at least briefly discussed these novel findings in the discussion.

References:

Lacombe J, Ferron M. Vitamin K-dependent carboxylation in β-cells and diabetes. Trends Endocrinol Metab. 2024;35(7):661-73 <https://pubmed.ncbi.nlm.nih.gov/38429160/>

Xie Y, Li S, Wu D, Wang Y, Chen J, Duan L, et al. Vitamin K: Infection, Inflammation, and Auto-Immunity. J Inflamm Res. 2024;17:1147-60 <https://pubmed.ncbi.nlm.nih.gov/38406326/>

3. Scientific Impact:
The paper provides valuable insights into how DMT1 patients may experience altered coagulation and increased sensitivity to anticoagulants. The finding that vitamin K may play a role in coagulation disorders within DMT1 patients has significant potential for both clinical applications and further research.
The study also opens the door for exploring the relationship between lipid profiles, vitamin K, and anticoagulation therapies in DMT1 patients. This could have important implications for managing cardiovascular risks in these patients.

Response: We are thankful to the reviewer for this encouraging comment.

4. Questions for Authors and Further Research:
On Vitamin K and DMT1: What mechanisms do you propose for the observed lower levels of vitamin K in DMT1 patients? Could this be linked to altered dietary intake, absorption, or metabolic processing of vitamin K in these individuals?

Response: We commented on this finding above. We cannot offer a plausible explanation for this phenomenon. Vitamin K is mainly present in HDL fraction, and our DMT1 patients present very similar lipoprotein levels as healthy donors, therefore, the differences in lipoproteins do not seem to be responsible. As far as we know, there is no data on differences in vitamin K intake, absorption, or metabolism between healthy people and DMT1 patients, but diabetic patients might simply need more vitamin K for controlling glycaemia. See please also our answer No.2.

On Lipid Profiles: The study found a paradoxical better lipid profile in DMT1 patients. Can you discuss in more detail how lipid metabolism in DMT1 may influence the coagulation system? Are these findings consistent with other studies, and how might they impact the broader therapeutic approach for managing DMT1?

Response: We looked at available literature and added information on the impact of lipids on blood coagulation and coagulation tests (see please the novel discussion). Even if these data are not specific to DMT1, they might bring another explanation.

Used references:

Kim JA, Kim JE, Song SH, Kim HK. Influence of blood lipids on global coagulation test results. Ann Lab Med. 2015;35(1):15-21.

Mutanen M, Freese R. Fats, lipids and blood coagulation. Current Opinion in Lipidology. 2001;12(1):25-9

Zwaal RF, Comfurius P, Bevers EM. Lipid-protein interactions in blood coagulation. Biochim Biophys Acta. 1998;1376(3):433-53.

On Anticoagulant Use: While the study shows increased sensitivity to direct anticoagulants in DMT1 patients, what are the potential clinical risks or benefits of using these anticoagulants in this population? Do the authors suggest any modifications to current anticoagulant dosing practices for DMT1 patients, considering their altered coagulation profiles?

Response: This is an interesting and clinically relevant point. In principle, our paper pointed out differences in coagulation based on BMI, inflammation, vitamin K, and cholesterol level. We do not think that some additional drug is needed, contrarily, we think that patients with abnormal values (very high BMI, high inflammatory state, low or high vitamin K and cholesterol levels) should be controlled about the status of their hemostastasis and then, eventually, the dose of their anticoagulant medication need to be modified. We have stated this newly as a conclusion of our study.

On Age and BMI Effects: How do the effects of age and BMI on coagulation profiles compare to other factors such as lipid profiles or medication adherence in DMT1 patients? Could these factors confound the results, and how might they be better controlled in future studies?

Response: Based on this comment, we performed an additional analysis of our data. There was almost a correlation between BMI and age (r=0.19; p=0.053), but no correlation of BMI to LDL-cholesterol (p=0.12) was observed, which is quite logical as many patients were treated with hypolipidemics. BMI (as well as age), however correlated with serum neopterin - see our new Figure 4, and we suppose that inflammation has the effect on blood coagulation. Concerning the second question, future studies should also include other inflammatory markers (e.g., interleukins 1, 6), and this is also our plan for future testing.

Literature Gaps: There seems to be a lack of studies directly comparing coagulation in DMT1 patients with those of other diabetic subtypes (e.g., type 2 diabetes). Could the authors expand on the differences or similarities in coagulation patterns between DMT1 and type 2 diabetes, citing other relevant research?

Response: Thank you for emphasizing. We have looked at available papers and found some relevant studies. We added some comments on the differences in the discussion.

Used references:

Ebrahim H, Asrie F, Getaneh Z. Basic Coagulation Profiles and Platelet Parameters Among Adult Type 1 and Type 2 Diabetes Patients at Dessie Referral Hospital, Northeast Ethiopia: Comparative Cross-Sectional Study. J Blood Med. 2021;12:33-42.

Oyakhire F, Adejumo BIG, Aiyegbusi OV, Ogie EO, Egho EV, Ochannah PB, et al. Coagulation Profile In Diabetes Mellitus And Its Association with Microvascular Complications in Uncontrolled and Controlled Diabetes at Edo Specialist Hospital, Benin-City, South-South, Nigeria. Medical Laboratory Technology Journal. 2023;9(1):11-21

Sobczak AIS, Stewart AJ. Coagulatory Defects in Type-1 and Type-2 Diabetes. International Journal of Molecular Sciences. 2019;20(24):6345

Stegenga ME, van der Crabben SN, Levi M, de Vos AF, Tanck MW, Sauerwein HP, et al. Hyperglycemia stimulates coagulation, whereas hyperinsulinemia impairs fibrinolysis in healthy humans. Diabetes. 2006;55(6):1807-12.

5. Final Recommendation:
The manuscript presents an intriguing and relevant exploration of the coagulation profiles in type 1 diabetes mellitus patients, particularly focusing on the role of vitamin K and its impact on anticoagulation sensitivity. However, I recommend addressing the minor grammatical issues and providing additional clarity on the mechanisms behind the findings. Expanding the discussion on the clinical implications of these results, particularly for anticoagulant treatment in DMT1 patients, would enhance the manuscript's impact.

Response: We have addressed all points raised by the reviewer, minor grammar issues were checked by the program Grammarly (<https://www.grammarly.com/>) and hopefully, our answers filled his/her expectations.

**Reviewer #5**: The study by Carazo A et al, titled “Type 1 diabetes mellitus patients had lower total vitamin K levels 2 and increased sensitivity to direct anticoagulants” aims to evaluate the importance vitamin K/ lipids in Type 1 diabetic coagulopathy. They have shown that anticoagulant treatment increases the coagulation time in patients with diabetes mellitus. While I find this finding intriguing, I have some concerns about the article.

Table 1: I wondered why the author did not measure glycated hemoglobin in healthy donors.

Response: We acknowledge the relevance of the comment raised. Glycated hemoglobin is not routinely measured clinically in samples from healthy patients, and therefore, we did not record this value for our healthy controls.

Author treated plasma with glucose and measured aPTT, PT, and TT. It would be interesting to treat plasma with triglycerides and cholesterol, and then measure the same parameters (aPTT, PT, and TT).

Response: This comment is very relevant and insightful. We also have data on hyperlipidemic patients, and it would be a really good idea to test the impact on different lipids on coagulation *in vitro,* as no complex study has been performed as yet. This testing will require substantial time, and we are not able to bring it in the deadline given by the editor.

What is the D-dimer concentrations in healthy donors compared to patients with diabetes mellitus? It would be worthwhile to correlate glucose, glycated hemoglobin, LDL, HDL, and TAG levels with D-dimer concentrations.

Response: We agree that this correlation would be very interesting. We measured a relatively large spectrum of different markers, but we have not included D-dimer. We are unable to do it retrospectively. We have specified it as a limitation of our study.

Table 2: I am interested in understanding how the external addition of heparin or Factor Xa inhibitors affects the r/p-value compared to DMSO alone (with glucose).

Response: Thank you for pointing it out. This is a very interesting question, but we are not able to provide a definite answer. If we look at basal values of INR (PT) and aPTT, the majority of PT/aPTT measured values both in the generally healthy population as well as in diabetic persons were in the physiological range; for this reason, correlation analysis did not bring a significant result. The physiological range is 0.8-1.2, and the highest value was 1.19 in our data set, and hence 100% were in the physiological range. In case of aPTT, the manufacturer range is 29 to 42 seconds; 5 generally healthy controls (16%) had shorter (2; 4%) or longer aPTT (3; 6%), while 4 (8%) of diabetic patients were not in the limit (longer). We also added the limits in the graph (Figure 1). If we look at the prolongation of PT by the most active compound (rivaroxaban), there was an increase by 165 % in the case of generally healthy controls and by 210 % in diabetic patients *vs.* the solvent. Contrarily, the increase in aPTT values was lower; dabigatran, the most active compound in case of aPTT, increased aPTT by 82% and 120%, in healthy and diabetic persons, respectively, *vs.* the solvent. It seems that, in general PT test was more sensitive as it gave more correlations and differences. Discussion has been changed to modify this aspect.

Authors should specify the type of statistical analysis used in the figure legend.

Response: We have amended each figure legend accordingly.
